# Resource management framework using simulation modeling and multi-objective optimization: a case study of a front-end department of a public hospital in Thailand

**DOI:** 10.1186/s12911-022-01750-8

**Published:** 2022-01-12

**Authors:** Tanatorn Tanantong, Warut Pannakkong, Nittaya Chemkomnerd

**Affiliations:** 1 Thammasat Research Unit in Data Innovation and Artificial Intelligence, Department of Computer Science, Faculty of Science and Technology, Thammasat University, Pathum Thani, Thailand; 2 School of Manufacturing Systems and Mechanical Engineering, Sirindhorn International Institute of Technology, Thammasat University, Pathum Thani, Thailand

**Keywords:** Resource management, Discrete event simulation, Patient satisfaction, Multi-objective optimization, Public hospital

## Abstract

**Background:**

The overcrowded patients, which cause the long waiting time in public hospitals, become significant problems that affect patient satisfaction toward the hospital. Particularly, the bottleneck usually happens at front-end departments (e.g., the triage and medical record department) as every patient is firstly required to visit these departments. The problem is mainly caused by ineffective resource management. In order to support decision making in the resource management at front-end departments, this paper proposes a framework using simulation and multi-objective optimization techniques considering both operating cost and patient satisfaction.

**Methods:**

To develop the framework, first, the timestamp of patient arrival time at each station was collected at the triage and medical record department of Thammasat University Hospital in Thailand. A patient satisfaction assessment method was used to convert the time spend into a satisfaction score. Then, the simulation model was built from the current situation of the hospital and was applied scenario analyses for the model improvement. The models were verified and validated. The weighted max–min for fuzzy multi-objective optimization was done by minimizing the operating cost and maximizing the patient satisfaction score. The operating costs and patient satisfaction scores from various scenarios were statistically compared. Finally, a decision-making guideline was proposed to support suitable resource management at the front-end departments of the hospital.

**Result:**

The three scenarios of the simulation model were built (i.e., a real situation, a one-stop service, and partially shared resources) and ensured to be verified and valid. The optimized results were compared and grouped into three situations which are (1) remain the same satisfaction score but decrease the cost (cost decreased by 2.8%) (2) remain the same satisfaction score but increase the cost (cost increased up to 80%) and (3) decrease the satisfaction score and decrease the cost (satisfaction decreased up to 82% and cost decreased up to 59%). According to the guideline, the situations 1 and 3 were recommended to use in the improvement and the situation 2 was rejected.

**Conclusion:**

This research demonstrates the resource management framework for the front-end department of the hospital. The experimental results imply that the framework can be used to support the decision making in resource management and used to reduce the risk of applying a non-improvement model in a real situation.

## Background

At present, healthcare services are an important service for people. Individuals seek a better quality of life, therefore the demand for using healthcare services is raised [1, 2]. The increase in the number of patients has led to the overcrowded problem which is the cause of long waiting times in healthcare service [3]. More importantly, waiting time is a significant performance measure that impacts patient satisfaction and loyalty to the service [4, 5]. The factors that cause the problem are high workload level, insufficient work procedure, interaction among workers problem, and adequate facilities availability [6]. According to World Health Organization, the standard ratio between number of medical doctors and number of patients should be 1:1000 [7]. The long waiting time problem often occurs in public healthcare services more than in private healthcare services [8]. Especially in developing countries such as India, Nigeria, and Vietnam, people are facing the patient overload problem in public hospitals [9, 10]. Also, in Thailand, the long waiting time is the main problem in the public hospital. The problem causes by the lack of resources and inefficient management. According to the statistic from the Ministry of Public Health and National Statistical Office of Thailand in 2018, the ratio between doctors and patients is 1:1771 which exceeds the standard. Lailomthong and Prichanont [11] pointed out that the long waiting time problem in Thailand public hospitals was caused by ineffective management of the queue system. The researchers proposed the new arrangement of the queue system, and it resulted in the reduction of waiting times without adding more resources.

Thammasat University Hospital (TUH) is a large and well-known public hospital in Thailand. TUH provides the service to over 1.1 million outpatients per year. As a large and educational hospital, it yields not only general treatments but also specific treatments such as heart treatments, eye treatments, dental treatments, physical therapies, and mental therapies. Because the hospital has many service departments, the system inside is complex in terms of data sharing. Moreover, the patients have different priorities not only for the level of symptoms but also for their privileges provided by the government, insurance, and the hospital. The problem of long waiting time is clearly shown in the beginning process of the hospital which is at the triage department and medical record department (MRD). These two departments are usually called together as a front-end department.

Many researchers proposed several methods to reduce waiting time by improving the healthcare system. One of the widespread techniques is discrete event simulation (DES). Over the past decades, DES has been used to model the patients’ behavior and promised a trustworthy outcome [12–14]. Especially, it is used to evaluate the change in outpatient department before the implementation in the real situation [15]. As demonstrate, inefficient resource management leads to long patient waiting time. DES is proved to help manager to better manage the human resource in outpatient department in order to reduce patient waiting time and total length of stay [16, 17]. Moreover, the simulation is also applied with optimization techniques for finding the best solution under specific conditions to decrease the waiting time. The changed waiting time can be referred to as the change in operating cost and patient satisfaction [18, 19].

From the above reasons, they lead to the objective of this research. The simulation model is built from collected data and optimized to minimize operating costs and maximize patient satisfaction score as the objectives. The paper is structured as follows: background, related work, methodology, experimental settings and results, and conclusion.

## Related works

### Patient satisfaction assessment

Patient satisfaction is defined mostly as an attitude of the patient toward care [20, 21] and represents a key point of communication and health-related behavior [22]. The patients who are satisfied with the service appear to be loyal to the health center and participate willingly with their treatment [23]. Patient satisfaction provides an opportunity for improvement in strategic decision making that meet patients’ expectation [24, 25]. Finding patient satisfaction has raised the popularity of using questionnaires as a tool for collecting data that supported by 37 reviewed studies by Almeida [26]. Al-Abri and Al-Balushi [27] stated that a patient satisfaction questionnaire is a significant quality improvement tool. An interview is also a considering method [20, 28, 29]. Boudreaux and O’Hea [30] reviewed over 50 studies on patient satisfaction in the emergency department. The data assessment method was conducted through the survey such as on-site surveys, via telephone after several days, and by paper and pencil in the department before leaving.

Larsen et al. [31] had concluded the problems of using satisfaction data and enhanced the usefulness of satisfaction data. The problems included the overestimated rate of satisfaction, lack of meaningful comparison bases, lack of standard scales, and difficulty to avoid biases. This research identified the way to achieve a useful satisfaction assessment method. Moreover, several strategies were recommended such as a focus on dissatisfaction data, use time-series analyses, and triangulate the measurement of satisfaction. Shirley and Sanders [32] also suggested the factors related to improving patient satisfaction which are physician-patient communication, the setting of appropriate expectations, minimizing waiting time, and provision of continuity of care. Additionally, Thompson et al. [33] supported that perceived waiting time affected patient satisfaction. The conclusion is that the long waiting time is caused the lower patient satisfaction [34, 35].

Using a questionnaire, however, the obtained result only provides the descriptive summary and cannot effectively predict patient satisfaction because of the uncertainty of patients. To overcome the problem, applying simulation provides the output that can be used to numerically predict patient satisfaction. Fan et al. [36] suggested a way to compute satisfaction by using the number of patients who abandoned the queue to represent dissatisfaction. The number of abandoned patients was proved and used as a quantitative evaluation index to measure patient satisfaction in the simulation model. However, it was not representing patient satisfaction.

Therefore, the method finding from Fan et al. [36] cannot be used. Our research is interested in using patient satisfaction to help manage the hospital. The method of converting the total time, which patients spent before seeing a doctor into a satisfaction score, is proposed with an in-depth interview with nurses about the relationship between length of stay (LOS) and patient satisfaction.

### Discrete-event simulation (DES)

In particular, the healthcare system is a system that is difficult to physically change according to continuous operations, limited resources, and high investment costs. A simulation model is a choice of imitating the system which can show the inside process of the system and identify a hidden problem [37]. Moreover, the improvement can be done easier in the simulation model than in the real system [38]. DES is used increasingly over the past decades. Leemis and Park [39] explained DES as a stochastic model (containing randomness in the model), a dynamic model (time-dependent), and a discrete model (state changing only when an event occurs). Bhattacharjee and Ray [40] studied the performance of a patient modeling method and specified the conditions of the event to use DES. First, patient flows are complex that includes many stages, classes of patients, patient priorities and routing. Second, when short-term analysis of system is considered. Last, DES is suitable for capacity planning, resource allocation and scheduling.

Many researchers use DES as a system modeling tool. Kovalchuck et al. [41] created a DES model representing patients’ flow across the departments in the hospital. Data mining was also applied to find the parameters input (i.e. surgery time, length of stay, and department path) for which it created the diversity of patients’ flow. The model successfully helped improve the patient's length of stay. Devapriya et al. [42] applied the data from electronic health records in DES to model bed allocation policies. The obtained model strongly represented the policy and helped forecast the number of patients, length of stay, and occupancy rate. Luo et al. [43] studied the queueing problem in the Computed Tomography (CT) examination department due to the difference in patients' priorities. DES was employed to solve the problem. The result showed that the model can provide a dynamic priority and reduce the waiting time of low priority patients. Bartos et al. [44] applied DES with Auto-Regressive Integrated Moving Average (ARIMA) for forecasting the model inputs instead of using distribution to generate randomness. The model was able to maximize the utilization of the resources.

Kritchanchai and Hoeur [45] built two DES models of old and new OPD to compare its performance and the result showed that if the healthcare changes the allocation of facilities as in the new OPD, the congestion in floor area can be reduced. Kalwar et al. [46] used DES to help in finding the optimal number of doctors in OPD by applying scenario analysis. Moreover, DES can be integrated with other techniques to improve the model performance. Kittipittayakorn and Ying [47] provided the reliable result using DES with agent-based simulation to reduce patient waiting time by approximately 32% from modifying the OPD processes in the model. Baril et al. [48] used Kaizen, which is the continuous improvement method, as the concept of how to improve the healthcare system. Then, using DES modeled the ideas and the result showed reducing in patient waiting time by 74% after 19 weeks of implementing.

Furthermore, an emergency department is a popular department that is used the simulation model for improving. Gul and Guneri [49] reviewed research studies on the topic of emergency department simulation. For more than 100 research studies, 95% of the studies were using DES. The main focuses were on an improvement in length of stay, resource utilization, and financial analysis of the department. Lin and Chia [50] introduced the technique, ARIMA, for forecasting the patient arrival in the emergency department together with DES. The result demonstrated a decrease in waiting time according to the doctor's rosters optimization. The most preferred software was identified to be an Arena software along with Simul8.

From all the above, DES is a popular method to build the simulation model for healthcare services in hospital departments. The characteristics of DES that is suitable to the situation of the hospital will help to create a more realistic simulation model. Therefore, this research conducts the hospital simulation with the DES method.

### Optimization

Optimization is an approach for finding the best solution related to the objective functions and constraints. The method is widely adopted to improve hospital management [51, 52]. The common objective functions aim to minimize overflow rate [53], minimize waiting time [54–56], and minimize the length of stay [57, 58].

Furthermore, the complexity of the hospital also affects the objective functions. Many considerations have been evaluated together such as operating costs, resource utilization, and patient satisfaction. Bouajaja and Dridi [59] concluded that there is still a few research that consider multiple objectives even though the real-world application is multidisciplinary. Thus, the use of multiple objective functions has been introduced to the healthcare system. Petering et al. [60] built a simulation of the intensive care unit (ICU) department. The research was focused on the total time patients spent in ICU and total time doctors spend on the treatment to find the reimbursement of the department. The objective function was to maximize the annual profit of the department. Not the only benefit was considered, but also the dissatisfaction of the patient was needed to be reduced. Fan et al. [36] built the simulation model with two objective functions which were maximizing benefit and minimizing dissatisfaction. Aliyu et al. [61] used multi-objective optimization to minimizing patient average waiting time as well as maximizing the doctor’s utilization in appointment scheduling in OPD. The process was done by the simulation model and the results were compared among current and improved scheduling. Chen and Wang [57] solved the resource allocation problem such as number of doctors, nurses, and other medical equipment in ED. The objectives were minimizing the expected patient length of stay, minimizing medical resource costs, and maximizing resource utilization rate. Using multi-objective stochastic optimization model was able to trade-off between the objectives. Also, Chang and Zhang [62] used weighted multi-objective optimization to manage the queueing system of inpatient waiting for bed. The optimized between patient admission rate and bed occupancy rate proved that the index value to evaluate hospital performance can be achieved at higher level.

As the literature above, the hospital system is usually large and complicated because of various numbers of departments and operations. The goal of operating health system is to operate with optimized cost while maintaining good service quality. Therefore, multiple aspects should be considered. Applying multiple objectives optimization is more practical to the hospital system. Therefore, the operating cost and patient satisfaction score are both considered in this research.

### A comparison study of simulation-based optimization

The reviewed related research is summarized in Table 1. This framework study is aimed to build a simulation model of the front-end department and applying multi-objective optimization in order to improve the resource management. The objectives are minimizing the operating cost and maximizing patient satisfaction.

**Table 1:** Summary of related work

References	Optimization	Department	Objective	Result
Fan, et al. [36]	Multi-objective	OPD	Max benefit & Min dissatisfaction	More effective outpatient scheduling
Wang et al.[53]	Multi-objective	Inpatient	Min overflow rate & waiting time & cost associate	Identify the process improvement and reduce waiting time
Chen et al. [54]	Single-objective	OPD	Min average waiting time or Max revenues of both hospital	The best feasible number of referral patients as a guideline for hospital collaboration
Chen and Lin [55]	Single-objective	OPD	Min average waiting time	Solved the problem of insufficient of medical resources and reduce waiting time
Ibrahim et al. [56]	Single-objective	ED	Min average patient waiting time	Optimized number of resources improve ED effiecncy
Chen and Wang [57]	Multi-objective	ED	Min expected length of stay, Min medical resource cost and Max resource utilization	New optimization approach to manage the medical resource allocation
Keshtkar et al. [58]	Single-objective	ED	Min patient length of stay	Improve in length of stay within the buget constraint
Petering et al. [60]	Single-objective	ICU	Max annual profit	Higher profit but high early dischage and re-enter
Aliyu et al. [61]	Multi-objective	OPD	Min average waiting time and Max doctor’s utilization	Better appointment system
Chang and Zhang [62]	Multi-objective	Inpatient	Max patient admission rate and Min bed occupancy rate	Hospital performance is higher
This frame work study	Multi-objective	Front-end department	Min operating cost and Max patient satisfaction	The decisión guideline to manage resource to improve patient satisfaction and operating cost

## Methodology

**Fig. 1:**
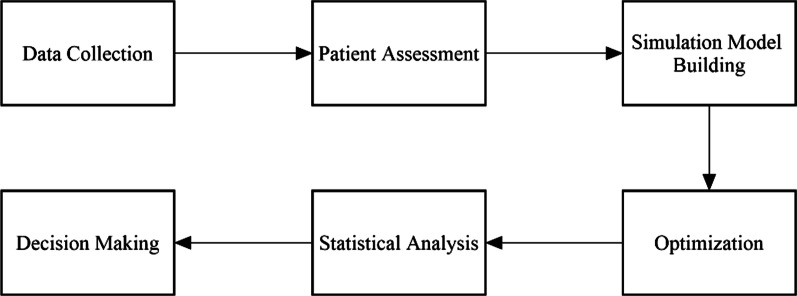
The components of the proposed framework

This section describes the overall processes of the proposed framework as shown in Fig. 1. First, the method of data collection which identifies the characteristics of the data and type of data collected is described. Then, the computational method of patient satisfaction assessment is given. Next, the overview of the simulation model and the details of the three patients' flow scenarios are described. The three patients’ flows are a current situation of the patients’ flow, a proposed patients’ flow for a one-stop service scenario, and a proposed patients’ flow for a partially shared resources scenario. Then, the optimization framework, which is integrated into the simulation model, is explained. Finally, the computational method of statistical analysis and the decision-making technique is explained.

### Data collection

**Fig. 2:**
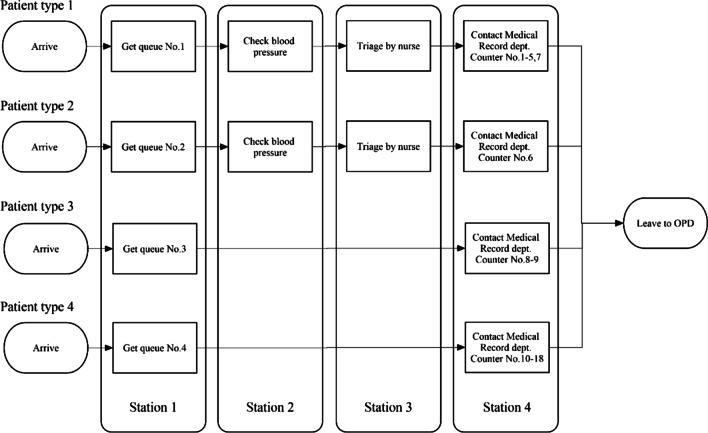
Flow path of each patient type

This research focuses on the front-end department of TUH covering the processes started when outpatients enter the hospital, and ended when patients leave the front-end department to an outpatient department (OPD). The processes during office hours, Monday to Friday 7:00–15:00, are considered. The queue ticket machine is started the operation at 5:00 and is closed at 14:50. The patients are divided into four types: (1) patients who do not have an appointment, (2) patients who are staffs or student of Thammasat University, (3) patients who are non-registered government or state enterprise officer privileges or patients who request to change their profile information, and (4) patients who have privilege from the government (e.g., universal health coverage). The patients’ flow paths are shown in Fig. 2. The patients’ flows of the first and the second types are: getting a queue ticket, checking blood pressure, triaging by nurses, filling information (only for new patients), contacting a medical record (MR) counter, and leaving to OPD. The patients’ flows of the third and the fourth types are: getting a queue ticket, contacting the medical record (MR) counter, and leaving for OPD.

The information was collected from April to May 2019. The collected data consists of timestamp at each station (shown in Fig. 2), the salary of nurses and operators, and patients' satisfaction questionnaire responses from senior nurses.

#### Timestamp data

To construct the simulation model, an interarrival rate of patients and operation time in the front-end department are the main inputs of the model. They were extracted from timestamps at each station. In observation, the timestamps of various patient events, including the queue ticket receiving, the station arrival, and the station leaving, are recorded. This data is used to compute the distributions of the interarrival rate and the operation time.

#### Hospital operating cost

Hospital operating cost is the cost of working operators that is computed from the base salaries of the nurses and hospital operators in the front-end department of TUH. The salary information is obtained from interviewing with department chiefs and nurses along with the amount of working time. The nurse and hospital operator are working on an average of 10 hours/day and 6 days/week. Due to the change in the number of operators over time, the hospital operating cost is computed to be Baht/hour.

### Satisfaction assessment method

The relationship between patient satisfaction scores and LOS is obtained from interviewing five experts who are the department chief and four senior nurses. The experts were asked questions related to the LOS and patient satisfaction scores. The patient satisfaction data is annually collected from patients by the hospital staffs. Therefore, the answer from the experts is related to the actual perception of patient satisfaction. The questions are the average time spending and the satisfaction score of each patient type, the spending time that would likely get the maximum satisfaction score, and the longest spending time with its satisfaction score. The score is given relatively to the LOS of the patient which is the time recorded when the patient enters the system until the patient leaves the system. Table 2 shows the data of satisfaction scores in relation to the LOS. The range of satisfaction score is between 0 and 100. Each patients’ type gives the optimistic value, the most likely value, and the pessimistic value of the satisfaction score related to LOS expectation. The satisfaction score data fits the triangular distribution (“Appendix 1”) which is the estimating method that involves professional’s opinion in the estimated task [63]. The method of converting LOS into satisfaction score is explained as follows. First, the recorded LOS is standardized by LOS’s mean and standard deviation (SD). Then, the standardized value is converted into a satisfaction score by satisfaction's mean and SD. The mean and SD of LOS, and satisfaction scores are computed from the collected data.

**Table 2:** The satisfaction score and LOS of each patients’ type

	Optimistic	Most likely	Pessimistic
**Patients’ type** 1			
LOS (min.)	55	90	120
Score (%)	100	75	50
**Patients’ type** 2			
LOS (min.)	15	30	60
Score (%)	100	75	50
**Patients’ type** 3			
LOS (min.)	45	70	90
Score (%)	100	75	50
**Patients’ type** 4			
LOS (min.)	45	70	90
Score (%)	100	75	50

### Simulation model construction

**Fig. 3:**
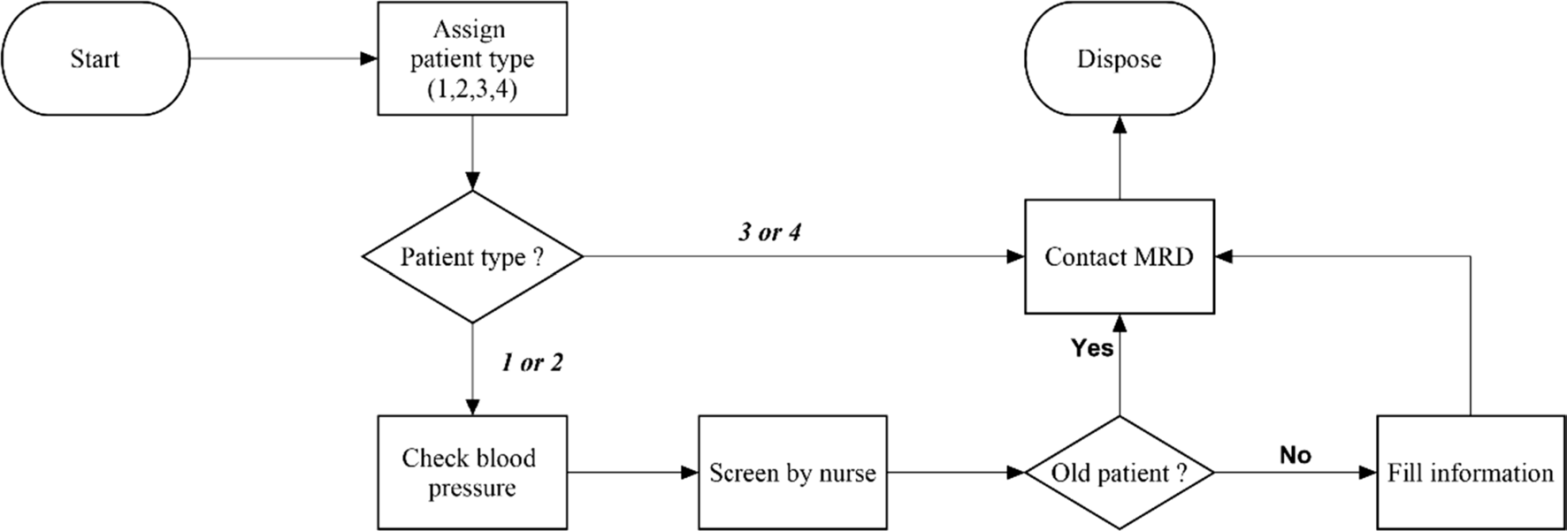
A patients’ flow logic in the simulated model

The DES model is built in Arena Simulation Version 16.0 (“Appendix 2”). Figure 3 shows the patients’ flow logic in the model. Initially, the model creates an entity representing an outpatient that come to the hospital. Next, the entity is identified information including a patients’ type, arrival time, and a patient status (i.e. old or new). The first decision is made to decide the path of the entity. The first path is for the patients’ type 1 and type 2 and the second path is for the patients’ type 3 and 4. The first path can be described as follows: checking blood pressure, triaging by a nurse, filling information if the entity is a new patient, contacting MRD, and dispose of the model. The flow of the second path is that the entity contacts MRD directly and disposes of the model.

The model operates under an uncertain environment (i.e. number of patients in each type, arrival rate, and operation time) which is assigned to the entities. An Input Analyzer program is used for analyzing the collected data to fit distributions representing the uncertainties of the system as demonstrated in Tables 3, 4, and 5. Patient is created and assigned the type according to the probability shows in Table 3. According to the simulation program, the patient arrival rate, which is the number of patients arriving per time unit, is converted into the interarrival time, which is the time between arriving patients. The distribution is presented in Table 4 which is used the exponential distribution. The distribution of operation time at each station is shown in Table 5.

**Table 3:** The probability distribution of each patients’ type in each period

Patients’ type	5:00–7:00	7:00–9:00	9:00–11:00	11:00–13:00	13:00–15:00
1	36%	35%	27%	26%	26%
2	10%	10%	9%	10%	10%
3	8%	11%	10%	10%	10%
4	46%	44%	54%	54%	54%

**Table 4:** The interarrival time of the patient during each period

Period	Distribution	Parameter (min)	Constant (min)	Expression
5:00–6:00	Exponential	Mean = 10	–	EXPO (10)
6:00–10:30	Exponential	Mean = 1.24	0.5	0.5 + EXPO (1.24)
10:30–15:00	Exponential	Mean = 2.67	–	EXPO (2.67)

**Table 5:** The operation time at each station

Operation	Distribution	Parameter (s)	Constant (s)	Expression
Check blood pressure	–	–	49	49
Triage by nurse	Exponential	Mean = 71.2	25	25 + EXPO (71.2)
Fill new information	–	–	300	300
Operating at MR for patient type 1	Weibull	Beta = 47.4		
Alpha = 0.94	14	14 + WEIB (47.4,0.94)		
Operating at MR for patient type 2	Weibull	Beta = 47.4		
Alpha = 0.94	14	14 + WEIB (47.4,0.94)		
Operating at MR for patient type 3	Triangular	Min = 84		
Mode = 146.8				
Max = 251	–	TRIA (84,146.8,251)		
Operating at MR for patient type 4	Triangular	Min = 59		
Mode = 72.8				
Max = 197	–	TRIA (59,72.8,197)		

Due to the change in patient density over the time, Table 4 which is the interarrival time of patient is divided into 3 period from early stage, peak stage, and decreasingly stage. While the change in Table 3 which is the probability of each patients’ type cannot fit within the same period. Therefore, the time window of Table 3 is set in equally period.

The performance measure of the simulation is LOS categorized into each patients’ type. It is used for validating and verifying the model. In Fig. 3, the LOS is recorded from the time between creating (Start) and disposing (Dispose) entities.

This research performs three simulation scenarios which are a current situation model, a one-stop service scenario, and a partially shared resources scenario. Each scenario has a different queueing policy when patients enter MRD which is explained in the following subsections. The two proposed scenarios are set up from the recommendation of the executive committee and hospital staffs. The first proposed scenario is one-stop service. The resources are shared to all types of patients. In this case, the efficiently managing the resource will resulted in increase of the resources’ utilization and reduce the number of resources as they can serve to all type of patients. Another proposed scenario is partially shared resources. The patient can go to different resources’ type is the same type is fully occupied and others are available. For this case, the resource will be more utilized and can help in adjusting the number of resources in each type.

#### Scenario 1: The current situation model of operation in MRD

**Fig. 4:**
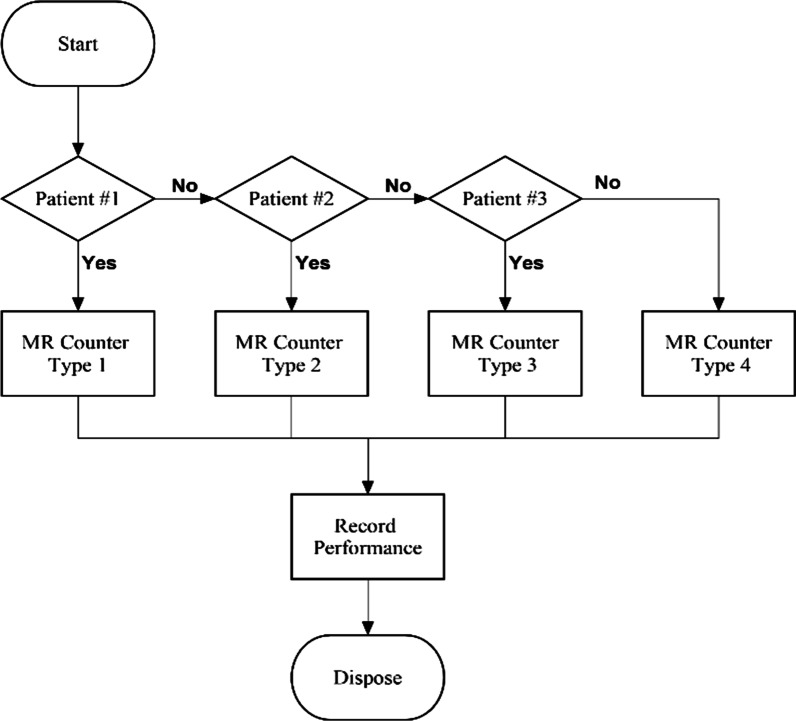
A flow logic of the current situation model in MRD

Figure 4 shows the flow logic when patients enter MRD in the current situation. After the entity (patient), which is already assigned a patient type, enters MRD, the decision is made to send the entity to only the MR counter dedicated to its patient type.

#### Scenario 2: Our proposed model for one-stop services

**Fig. 5:**
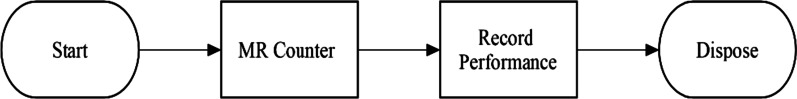
A flow logic of one-stop service model in MRD

In this scenario, MR counters work for various patient types as one-stop service counters. This model proposes that there is no need to separate the patient when entering MRD. The flow logic is depicted in Fig. 5. The uncertainty parameters, i.e., probability distribution of each patients’ type, interarrival rate, and operation time, are applied in the model as same as in the model of Scenario 1.

#### Scenario 3: Our proposed model for partially shared resources

**Fig. 6:**
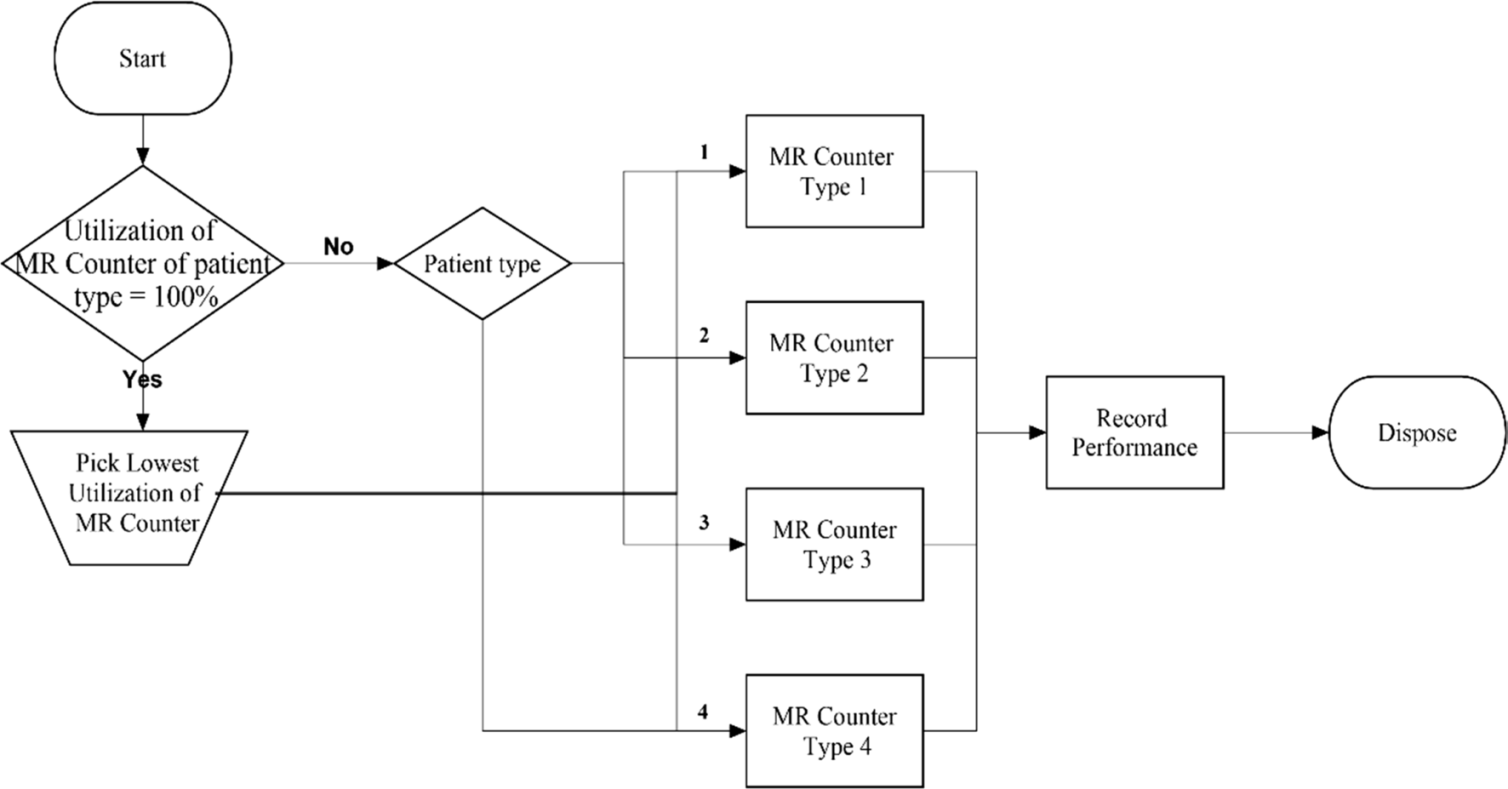
A flow logic of partially shared resources model in MRD

In this scenario, each MR counter type serves a patient who has the same type with the highest priority. However, when the patient cannot be served by the same type of MR counters because they are fully occupied, the patient will enter the other type of MR counter that has the lowest utilization. The flow logic is presented in Fig. 6. The uncertainty parameters, i.e., probability distribution of each patients’ type, interarrival rate, and operation time, are applied in the model as same as in the model of scenario 1.

#### Verification and validation

Model verification is conducted to confirm the correction of the assumptions and specifications of the model before model implementation [64]. The verification method is, first, to set the simulation model at an extreme case by letting the model creates one entity for each patient type and using constant operation times. The LOS of each patients’ type is considered as a performance measure. The LOS in manual calculation is calculated by sum all operation times together for each patient type. While LOS from simulation model comes from running the simulation model with constant operating time. Then, the LOS from running the simulation model is compared with the LOS calculated manually assuming no queue occurred. In comparison, the LOS from simulation and manual calculation must be equal for confirming the correctness of the model.

Model validation is to check the model’s accuracy of representing the real system. Naylor and Finger [65] proposed the widely used technique to validate the model. The model output to corresponding output in the real system was compared. The performance measure of the model, which is LOS, is compared with the LOS collected from the real system which has proven the correctness by the experts. Moreover, this study works under the supervised from experts throughout the process. In this study, a statistical comparison technique, i.e., t-test, is used. Two hypotheses are applied as follows: null hypothesis, H_0_: $\mu_{1} = \mu_{2}$, and alternative hypothesis, H_1_: $\mu_{1} \ne \mu_{2}$, when $\mu_{1}$ and $\mu_{2}$ represent the mean LOS from the real system and the simulation model, respectively. The meaning of the hypotheses is to test the similarity between ${\mu }_{1}$ and ${\mu }_{2}$. The testing result shows the *p* value to indicate the similarity. By setting percentage confidence at 95%, if the *p* value is equal to or greater than 0.05, the null hypothesis (H_0_) cannot be rejected. Then it implies that the current situation model is valid because ${\mu }_{1}$ and ${\mu }_{2}$ are similar as they are not statistically significantly different. Therefore, if the current situation model is valid, it can be used for further analysis and model extension in other scenarios. The test is done by a Minitab 18 program.

### Model optimization

To improve the performance of the simulation models, the model parameters (i.e., number of human resources at each station) are optimized by an optimization technique. In this research, there are two focused objectives which are minimizing LOS and maximizing patients’ satisfaction. The optimization technique is used to identify the optimal amount of resources relative to the objective functions. In the model optimization experiments, the models are optimized with single and multi-objective functions. The details are explained in the following sub-sections. The optimization experiments are done by OptQuest, a built-in application of Arena Simulation Software. It is a heuristic optimization approach. It searches within the decision variable range and run the simulation model with the decision variable setting to generate the result. After that, the result is checked its feasibility. If the result is feasible, the program will keep the result. Otherwise, the result will be rejected. Then, the program repeats the step from finding the decision variable. It will stop the optimization when there is no further improvement in objective value over a condition setting, in this research set to stop when no improvement result within 100 tries.

#### Single-objective optimization

Many researchers applied optimizations for minimizing waiting time or maximizing patients’ satisfaction. These objectives are focused on the patient side. However, on the hospital side, a minimizing cost is a main concern. Therefore, the two objective functions are proposed as follows: (1) minimizing a cost of operation and (2) maximizing a patient satisfaction score. The parameter notations and mathematical models of the two objective functions are defined below.

**Parameters:**

- *i*: Type of resources.
- *j*: Patient number.
- *t*: Operating period.
- *N*: Total number of patients.
- *A*: Total number of MR counters.
- *c*_*i,t*_: Operating cost of resource *i* in period *t.*

**Decision variable:**

- *P*_*j*_: Satisfaction score of patient *j.*
- *n*_*i,t*_: Number of resources *i* in period *t.*

From the discussion with executive committee, the concern about adjusting the number of resources used is raised. Therefore, this study focuses in using the number of resources as the model parameter. Moreover, using one model parameter (number of resources) has been proved that it can improve the system effectively [56–58].

For a single-objective optimization approach, each objective function is optimized separately with the identical set of constraints as follows:

**Objective function:**

1 $${\text{Minimize}}\,\,\,Z_{1} = \mathop \sum \limits_{t} \mathop \sum \limits_{i} \left( {c_{i,t} \times n_{i,t} } \right)\quad \forall {\text{i}} \in {\text{i}},\quad \forall t \in t$$

2 $${\text{Maximize}}\,\,\,Z_{2} = \frac{{\mathop \sum \nolimits_{j} P_{j} }}{N}\quad \forall j \in j$$

$${\text{Subject}}\,{\text{ to}}:$$

3 $$\mathop \sum \limits_{i = 3}^{i = 7} n_{i,t} \le A\quad \forall t \in t$$

4 $${\text{Pj}} \in \left[ {0, 1} \right]\quad \forall j \in j$$

5 $$n_{i,t} \ge 0, n_{i,t} \,\,\, {\text{is integer}}\,\,\,\,\forall i \in i, \,\,\,\,\forall t \in t$$

Two objective functions are formulated for minimizing the operating cost (1) and maximizing the average patient satisfaction score (2). In (1), the total operating cost is calculated from the operating cost multiplied by the number of resources in each operating period, then the summation of value is applied. In (2), the average patient satisfaction score is calculated from the summation of the satisfaction score of each patient divided by the total number of patients. The constraint (3) ensures the number of an active MR counter which cannot exceed the available capacity. The constraint (4) confirms the range of patient satisfaction score to be varied from 0 to 100%. The constraint set (5) restricts the negativity number of resources, and it must be an integer.

#### Multi-objective optimization

In a large hospital, a board of directors typically considers many aspects simultaneously in decision making. Therefore, presenting an optimized value from one objective may not cover all the aspects of the hospital. Multi-objective optimization is introduced by combining many objective functions and solving at the same time. The importance of each objective is different, thus it is set by adding the weight into the objective function [66]. Users can specify the weight on each objective based on their preference. However, occasionally, users have difficulties to transform their qualitative preference into a quantitative weight. In this study, the preferences from the hospital on cost and satisfaction objectives are not given. Hence, the weight set is from the sensitivity analysis recommended by Coello and Christiansen [67]. Five sets of weight settings are used. Each set contains the weight of cost objective and weight of satisfaction objective. The summation of all weights in each set is one. The detail of the weight setting is shown in Table 6.

**Table 6:** A weight setting for multi-objective optimization

Nos	Weight of cost	Weight of satisfaction
1	0.8	0.2
2	0.2	0.8
3	0.6	0.4
4	0.4	0.6
5	0.5	0.5

In this situation, the outcomes of all objectives are presented in different units which cannot be compared. Applying a fuzzy linear programming technique would eliminate the problem. Zimmermann [68] expressed objective values *Z*_*k*_, *k* = 1,…,p, by a fuzzy set. Each objective is formulated by separating into its maximum and minimum values from the single-objective optimization. The fuzzy set is given as follows:

6 $$f_{k} \left( n \right) = \left\{ {\begin{array}{*{20}c} {\frac{{Z_{k}^{ + } - Z_{k} }}{{Z_{k}^{ + } - Z_{k}^{ - } }}\quad for \,min \,obj.} \\ { \frac{{Z_{k} - Z_{k}^{ - } }}{{Z_{k}^{ + } - Z_{k}^{ - } }} \quad for\, max\, obj.} \\ \end{array} } \right.$$

The fuzzy set is defined by the difference between an objective value and its pessimistic value over the difference between its optimistic and pessimistic values. In (6), the value increases linearly from 0 to 1. When $f_{k} \left( n \right)$ is one, it indicates the optimistic case of the objective. In contrast, when $f_{k} \left( n \right)$ is zero, the pessimistic case of the objective is shown.

By combining weight and fuzzy sets, a weighted max–min method for a fuzzy multi-objective model was proposed by Lin [69]. The method is to make the ratio of an achievement level objective function ($\lambda$) as close to the ratio of the weight. In [70], the weighted max–min method is simplified and applied in this study as follows:

7 $${\text{Max}}\,\,\lambda$$

$${\text{Subject}}\,{\text{ to}};$$

8 $$w_{k} \lambda \le f_{k} \left( n \right)\quad k \, = {1}, \ldots ,{\text{p }}\left( {{\text{for}}\,{\text{ all}}\,{\text{ objective}}\,{\text{ functions}}} \right)$$

9 $$\lambda \in \left[ {0, 1} \right]$$

The objective is maximizing $\lambda$ (7) which is specified the range of values as in the constraint (9). The constraint (8) is the weight constraint on each objective *k* in which is limited by the fuzzy set. The weight, w_k_, is fixed as shown in Table 5. The step of solving optimization is, first, solving single objective optimization to get the optimistic and pessimistic value from both objective functions. Then, both value is used in normalized the fuzzy function in multi-objective optimization.

### Comparison among scenarios

After the simulation models are optimized in every set of weights, the average satisfaction scores are compared to demonstrate the increase in patient satisfaction. There are two statistical methods, which are the analysis of variance (ANOVA) and the Tukey's test, used for identifying the difference between sets of weights. ANOVA is suitable for analyzing the effect of a factor, which has more than two levels, on a response variable [71]. In this study, the factors are the cases (seven weights of satisfaction objective i.e., 0, 0.2, 0.4, 0.5, 0.6, 0.8, and 1), the scenarios (four scenarios i.e., 1, 2, 3 and current scenario without optimization), and the interaction between cases and scenarios. The replication is employed as a block. The response is the patient satisfaction score (i.e., a sequence number from 0 to 100). The hypotheses of the test are shown as follows: null hypothesis, H_0_: $\mu_{1} = \mu_{2} = \mu_{3} = \ldots = \mu_{i}$ and alternative hypothesis, H_1_: means are not equal, where ${\mu }_{i}$ represents the mean of satisfaction score in the *i*th level of the weight of satisfaction objective. The hypotheses are set to prove that whether the satisfaction score is statistically different among the levels of the weight of the satisfaction objective. If the p-value is less than 0.05, the null hypothesis will be rejected. The meaning is the satisfaction scores are different among the levels of the weight of satisfaction objective. Then, Tukey's test is used to indicate which the weight on the satisfaction objective gives the difference. The Tukey’s test is done for multiple comparisons to identifying which level is significantly different from others [72]. The method is to compare all possible pairs of satisfaction means. Then, the means that are not significant differences are grouped. Both statistical tests are done in a Minitab 18 program.

After the statistical analysis is done, the means of satisfaction scores of each situation are compared and the operating cost is considered at the same time. The decision is extracted from the discussion with the executive committee and hospital staffs as shown in Table 7. The satisfaction score and the operating cost are compared whether they are significantly higher, unchanged, or lower from the values of the current scenario without optimization.

**Table 7:** The decision guidelines

Nos.	Satisfaction score	Operating cost	Suggestion	Remark
1	Higher	Higher	Depend on the decision-maker	It gets higher satisfaction, and the cost may increase within the acceptable amount
2	Higher	Unchanged	Recommended	It gets higher satisfaction while spending the same amount of the cost
3	Higher	Lower	Recommended	It gets higher satisfaction and can lower the cost
4	Unchanged	Higher	Not recommended	There is no change in satisfaction but the cost increases
5	Unchanged	Unchanged	Depend on the decision-maker	There is no change in both factors. The decision-maker may consider other factors such as the satisfaction of workers
6	Unchanged	Lower	Recommended	There is no change in satisfaction, and it can decrease the cost
7	Lower	Higher	Not recommended	It not only decreases the satisfaction, but it also increases the cost
8	Lower	Unchanged	Not recommended	It spends the same amount of the cost, and the satisfaction still decreases
9	Lower	Lower	Depend on the decision-maker	Due to the cost reduction, the satisfaction score is lower but it is still acceptable

## Experimental settings and results

This section first describes the replication run parameter setting of the simulation model. Then, the verification and validation results of the simulation model are shown. Finally, the optimization results from the three applied scenarios are explained and discussed.

### Replication run parameter setting

In the simulation model, the number of replications, replication lengths and warm-up period are specified as a user specification. In this research, each replication in the model is represented as one working day. Therefore, the replication length is the period of operation starting until the last patient leaves the department. The model is set the terminating condition as after a specific time if the number of entering entities equals the number of leaving entities, the model will stop. The specific time is set at 600 min (10 h). The number of replications firstly uses 10 replications. Then the error is calculated according to the calculation method from Kelton et al. [73] and the error value is greater than the acceptable error (5%). The number of replications is recalculated. The new number of replications, which is eight replications, is used and satisfied the threshold. The warm-up period in this research is not added. Even though, warm-up period is commonly required in simulation model, however if the simulation model focuses in the starting of business or in the environment where it starts with empty entity such as airport or restaurant, the warm-up period can be neglected [74]. According to the environment of the hospital, the system starts with empty patient and the research focuses on how to manage the resources from the start of the day until the end of the day, not only during the steady-state period.

### Verification and validation

Verification is done by comparing LOS from manual calculation and simulation results. Table 8 shows the comparison of LOS of each patient type. There is no difference between the LOS from manual calculation and simulation results in every patient type. Therefore, the comparison result confirms the accuracy of the logic in the simulation model.

**Table 8:** Verification test by comparing two different computing LOS

Patient type	LOS calculated manually (min)	LOS from simulation without queue (min)
1	3.47	3.47
2	3.47	3.47
3	2.67	2.67
4	1.83	1.83

In the validation section, the average LOS of each patient type from the simulation model is compared with the average LOS from the data collection. The statistical technique, i.e., t-test, is used to test the difference between the two average LOS. P values of all queue types, as demonstrated in Table 9, are reported to be greater than 0.05. From these results, with a 95% confidence level, there is no strong evidence to reject the null hypothesis. In another word, it shows no difference between the two LOS. The result confirms that the simulation model can represent the real system and can be used for further analysis.

**Table 9:** Validation test by comparing LOS from the simulation with data collection

	Patients’ type 1	Patients’ type 2	Patients’ type 3	Patients’ type 4
Actual average (min)	56.47	25.52	38.89	53.86
Actual SD	41.90	25.93	22.38	34.90
N	167	79	46	73
Simulation average (min)	56.60	27.40	37.70	47.90
Simulation SD	27.20	20.90	20.40	27.60
N	3999	1158	1226	6244
*p* value	0.95	0.45	0.70	0.07
Replication error	1.49%	4.38%	3.02%	1.43%

### Results from the optimized simulation model

First, the current scenario without optimization is run under a similar setting as in the real system. The results are the operating cost of 5296.5 Baht/day and the average satisfaction score of 85.81%. To improve the operating cost and the average satisfaction score, the optimization is done by OptQuest. The processing time used to run the optimization is approximately 4–5 h for each run. There are three optimization scenarios. In each scenario, there are optimal solutions from seven cases: two solutions from the single-objective optimizations, and five solutions from the multi-objective optimizations. The objective values are the operating cost (Z_Cost_), the average satisfaction score (Z_Sat_), and *λ* only for the multi-objective optimization.

#### Optimization results of scenario 1: the current situation model of operation in MRD

Table 10 presents the optimization results. For the single-objective optimization, the weight of the interested objective equals one, and another equals zero. When the weight of the cost equals one, the optimization gives the lowest operating cost, 4521 baht/day, and the lowest satisfaction score, 45.9%. When the weight of satisfaction equals one, the optimization gives the highest satisfaction, 89.95%, and the highest operating cost, 6864 Baht/day.

**Table 10:** The obtained solutions from the scenario 1 model

Case	Optimization	W_Cost_	W_Sat_	$\lambda$	Z_Cost_	Z_Sat_
1	Single-obj	1	0	–	4,521	45.9%
2		0	1	–	6,864	89.95%
3	Multi-obj	0.8	0.2	1	4,966.5	67.2%
4		0.6	0.4	1	5,280	70.1%
5		0.5	0.5	1	5,676	88.1%
6		0.4	0.6	1	5,742	88.17%
7		0.2	0.8	1	5,940	88.2%

For the multi-objective optimization, within the interval of the weight setting, the results are shown that the operating costs are varied between 4966.5 and 5940 Baht/day, and the satisfaction scores are varied between 67.2 and 88.2%.

#### Optimization results of scenario 2: our proposed model for one-stop services

The optimized solutions of scenario 2 are shown in Table 11. The optimized operating cost, 2161.5 Baht/day, and the optimized satisfaction score, 31.2%, are shown when the weight of cost equals one. The optimized operating cost, 5940 Baht/day, and the optimized satisfaction score, 89.9%, are shown when the weight of satisfaction equals one.

**Table 11:** The obtained solutions from the scenario 2 model

Case	Optimization	W_Cost_	W_Sat_	$\lambda$	Z_Cost_	Z_Sat_
1	Single obj	1	0	–	2161.5	31.2%
2		0	1	–	5940	89.9%
3	Multi-obj	0.8	0.2	1	2755.5	40.4%
4		0.6	0.4	1	3630	61.2%
5		0.5	0.5	1	3844.5	63.7%
6		0.4	0.6	1	4422	79.7%
7		0.2	0.8	1	5148	88.1%

For multi-objective optimizations, the optimized operating costs are varied between 2755.5 and 5148 Baht/day, and the optimized satisfaction scores are varied between 40.4 and 88.1%.

#### Optimization results of scenario 3: our proposed model for partially shared resources

Table 12 demonstrates the optimized solution of the scenario 3. The optimized solution from the weight of cost equals to one shows the lowest operating cost, 3943.5 baht/day, and the lowest satisfaction score, 14.97%. While the weight of satisfaction equals one, the optimized solution gives the highest operating cost, 9174 baht/day, and the highest satisfaction score, 90.62%.

**Table 12:** The obtained solutions from the scenario 3 model

Case	Optimization	W_Cost_	W_Sat_	$\lambda$	Z_Cost_	Z_Sat_
1	Single obj	1	0	–	3943.5	14.97%
2		0	1	–	9174	90.62%
3	Multi-obj	0.8	0.2	1	4983	44.4%
4		0.6	0.4	1	5329.5	81.4%
5		0.5	0.5	1	6451.5	83.6%
6		0.4	0.6	1	6748.5	84.8%
7		0.2	0.8	1	7029	86.1%

For multi-objective optimization, the results are shown that the operating costs are distributed between 4983 and 7029 baht/day, and the satisfaction scores are distributed between 44.4 and 86.1%.

## Results and discussion

ANOVA and Tukey’s tests are performed to analyze the simulation results. Table 13 presents the result of ANOVA. The result shows that the interaction effect between cases and scenarios is statistically significant (*p * < 0.0001). Thus, the effect of the single factor is neglected. As the interaction effect between cases and scenarios is significant, the means of satisfaction score in all cases and scenarios are compared by Tukey’s test with the 95% confidence level.

**Table 13:** The results from comparing all cases and scenarios by ANOVA

	DF	Adj SS	Adj MS	F value	*p* value
Case	6	65,285	10,880.8	938.21	0.000
Scenario	3	17,597	5865.6	505.77	0.000
Replication	9	2331	259.0	22.33	0.000
Case*scenario	18	31,518	1751.0	150.98	0.000
Error	243	2818	11.6		
Total	279	119,549			

The results from Tukey's test are detailed in Table 14. The operating cost is also considered in the discussion. The values in the highlighted row are the results from the current scenario without optimization (scenario 9) which is employed as a benchmark in comparison with the other scenarios. After the comparison, the decision is made according to the guidelines in Table 7. The comparison results show that it can be group into three situations to match the guidelines which are guideline number 4, 6 and 9.

**Table 14:** Tukey's test result

Nos.	Experimental setting			Obtained results													
	Scenario	Case	Weight	Cost	N	Satisfaction mean	Grouping										
1	3	2	1	9174	10	90.626	A										
2	2	2	1	5940	10	89.913	A	B									
3	1	2	1	6864	10	89.898	A	B									
4	1	7	0.8	5940	10	88.192	A	B	C								
5	1	5	0.5	5676	10	88.161	A	B	C								
6	1	6	0.6	5742	10	88.144	A	B	C								
7	2	7	0.8	5148	10	88.110	A	B	C								
8	3	7	0.8	7029	10	86.089	A	B	C	D							
**9**	**9**	**1–7**	**0–1**	**5296.5**	**100**	**85.810**	**A**	**B**	**C**	**D**							
10	3	6	0.6	6748.5	10	84.832		B	C	D	E						
11	3	5	0.5	6451.5	10	83.584			C	D	E						
12	3	4	0.4	5329.5	10	81.388				D	E						
13	2	6	0.6	4422	10	79.709					E						
14	1	4	0.4	5280	10	70.144						F					
15	1	3	0.2	4966.5	10	67.242						F	G				
16	2	5	0.5	3844.5	10	63.699							G	H			
17	2	4	0.4	3630	10	61.170								H			
18	1	1	0	4521	10	45.982									I		
19	3	3	0.2	4983	10	44.380									I		
20	2	3	0.2	2755.5	10	40.379									I		
21	2	1	0	2161.5	10	31.216										J	
22	3	1	0	3943.5	10	14.969											K

The highlighted row (number 9) is the results from the current scenario without optimization

Among the three situations, the first and second situations include the scenarios and cases that are laid in group A to D same as the scenario 9. This indicates that their means of satisfaction score are not statistically different. For the first situation, the result of scenario 2 (one-stop service scenario) and the weight of satisfaction objective of 0.8. gives a cheaper cost. It relates to the guideline number 6 in Table 7. The suggestion is to recommend applying this situation for improving the system.

The second situation contains the remaining scenarios and cases that have higher costs. The situation refers to the guideline number 4 in Table 7 which suggests not to recommend applying this situation for improving the system.

The third situation involves the scenarios and the cases laid in the group E only (scenario 2 and case 6) and the groups F to K. They show a significantly lower mean of satisfaction score and cheaper cost than the scenario 9. The situation refers to the guideline number 9 in Table 7. The suggestion is to decide according to the decision-maker preference. If the lower satisfaction score is within an acceptable range and is worth the cost-saving, the scenarios and the cases should be selected.

Many possible scenarios and cases match the third situation. Therefore, the steps of selecting the best scenario and the best case to match the preference are explained as follows. First, the decision-maker selects the minimum score of patient satisfaction that the hospital prefers.

Then, from the grouping in Table 14, the group that contains the selected minimum score within its ranged are considered. If there is no group containing the selected minimum score, then the group having the nearest greater score is considered. Within the considered group, a case in a scenario that has the cheapest cost is selected. However, if the other groups having the higher satisfaction score contain cheaper cases, then the cheapest case among them is selected instead.

For example, assume the required minimum score of patient satisfaction is 70. The score of 70 presents in group F in Table 14. Then, in group F, the number 15 which has the cheapest cost is selected. After that, the above groups are considered to find whether there is any cheaper cost. Table 14 presents the number 13 that has a cheaper cost than the number 15. Hence, the number 13, which is the scenario 2 (one-stop service scenario) and the weight of the satisfaction objective of 0.6, is recommended to be chosen.

However, to select a suitable solution among the recommended solutions under the different situations (i.e., first and third situations), the hospital may need to prioritize the factors. If the patient satisfaction has a higher priority, the recommended solution under the first situation should be applied. In another case, if the budget has a higher priority, the recommended solution under the third situation should be considered. For example, in the case that the hospital plans to reduce the budget in other sections to support the work of the COVID-19 protecting section, the scenarios and the cases that are in the third situation will help the decision-maker to select the best model that can save the cost and remaining the patient satisfaction in a fair level.

The resource scheduling for each scenario and cases is shown in Table 15. The resources, which are nurses at triage department and MR operators, are scheduled in four periods. The sequence is set as same as in Table 14. For example, in number 1, which is scenario 3 case 2, the resources are scheduled during 7:00 to 9:00 as follows: 6 nurses at triage for patients’ type 1, 3 nurses at triage for patients’ type 2, 3 MR operators for patients’ type 1, 3 MR operators for patients’ type 2, 2 MR operators for patients’ type 3, and 10 MR operators for patients’ type 4.

**Table 15:** The resource scheduling result

Nos.	7.00–9.00								9.00–11.00								11.00–13.00								13.00–15.00							
	N	N2	M	M1	M12	M2	M3	M4	N	N2	M	M1	M12	M2	M3	M4	N	N2	M	M1	M12	M2	M3	M4	N	N2	M	M1	M12	M2	M3	M4
1	6	3		3		3	2	10	6	3		2		3	4	9	5	3		5		3	2	8	5	3		6		3	2	7
2	6	2	18						2	3	14						2	2	17						1	1	5					
3	4	1		5	1	1	2	9	4	1		4	2	1	3	8	3	1		2	1	1	2	10	4	1		5	1	1	5	4
4	3	1		5	1	1	2	9	3	1		4	1	1	3	9	2	1		2	1	1	2	10	2	1		1	1	1	5	4
5	3	1		5	1	1	2	9	3	1		4	1	1	2	10	2	1		2	1	1	2	10	2	1		1	1	1	1	4
6	3	1		5	1	1	2	9	3	1		4	1	1	2	10	2	1		2	1	1	2	10	2	1		1	1	1	1	5
7	4	1	18						6	3	8						6	3	1						1	3	1					
8	3	3		2		2	5	7	4	2		7		2	2	7	2	2		5		2	3	8	3	2		4		3	5	2
9	6	2		5	1	1	3	10	5	1		5	1	1	3	10	3	1		4	1	1	2	7	2	1		3	1	1	1	5
10	3	1		1		3	1	10	6	3		7		3	5	3	1	1		4		3	1	10	3	1		7		3	5	2
11	3	1		1		3	1	10	6	3		7		3	5	1	1	1		4		3	1	10	1	1		7		3	5	3
12	3	3		4		2	2	10	5	1		1		3	4	3	1	1		7		1	1	3	1	2		1		3	5	2
13	4	1	15						3	2	15						3	3	1						1	3	2					
14	3	1		5	1	1	1	10	1	2		3	1	1	1	10	3	1		1	1	1	1	10	1	1		2	2	1	1	1
15	6	1		7	1	3	1	5	1	3		7	3	3	4	1	1	1		1	1	1	5	1	1	1		1	3	3	1	1
16	3	1	13						1	3	8						2	1	11						1	1	1					
17	2	1	13						6	2	1						2	2	4						2	2	2					
18	6	1		7	1	3	1	5	1	3		7	3	3	4	1	1	1		1	1	1	5	1	1	1		1	3	3	1	1
19	3	2		6		2	1	7	4	1		1		2	3	2	1	2		1		2	2	2	3	2		6		2	4	1
20	2	2	11						2	1	10						2	1	1						1	1	1					
21	1	1	10						5	1	1						1	1	1						1	1	1					
22	1	2		1		2	1	1	1	2		4		1	2	3	2	1		4		1	1	1	1	1		7		1	2	6

N = Nurse who services patients’ type 1, N2 = Nurse who services patients’ type 2, M = MR operator for scenario2, M1 = MR operator who services patients’ type 1, M12 = MR operator who services patients’ type 1and 2, M2 = MR operator who services patients’ type 2, M3 = MR operator who services patients’ type 3, M4 = MR operator who services patients’ type 4

## Conclusion

This research has proposed the framework for supporting in decision making to solve queueing problems in the front-end department including the triage department and the medical record department with a case study of a public hospital in Thailand. Typically, public hospitals are facing overcrowded problems according to the lack of resources (e.g. doctors, nurses, and equipment). Moreover, the crowded patients lead to lower patients' satisfaction. It causes dissatisfaction and unwillingness to return to service. To overcome the problem, the patient length of stay must be reduced which causes in the increasing number of resources. The problem shows the relationship between the patients' satisfaction and the LOS. Therefore, this study proposes the method to convert the LOS to the satisfaction score by triangular distribution and optimization methods to find the number of resources that increase patient satisfaction significantly. In addition, the problem is also related to the budget that is insufficient and poor management.

The simulation model is developed along with the simulation-based optimization using OptQuest to find the optimal resources used in the department. The validation and verification methods are proposed to assure the model accuracy and the model validated. A weighted max–min for fuzzy multi-objective optimization is used to solve two objectives which are minimized the operating cost and maximized the patients’ satisfaction respected to the LOS. Moreover, scenario analysis is applied to the model for system improvement. The scenarios applied are called a one-stop service model and a partially shared resource model. The optimized solution is compared the difference in the mean of satisfaction score to the non-optimized solution by using ANOVA and the Tukey’s test.

In the case study, the comparison demonstrates no scenario and case that generates significantly higher satisfaction than the current scenario. It indicates that the front-end department works at a satisfactory level. However, two situations can be applied to improve the system in other aspects. The first situation is the one-stop service scenario with the weight of the satisfaction objective of 0.8. The situation statistically confirms that the operating cost is lower while the satisfaction remains at the same level as the current scenario. Another situation is that the operating cost is lower, and the satisfaction is also lower, but it is at the acceptable level from the hospital preference.

In addition to a managerial perspective, the results from our proposed simulation model can be used to reveal the impact of a new scenario in the hospital before actual implementation. A suitable scenario can be chosen from the situations that are optimized under multiple objectives and statistically prove to be better than the current scenario in the specified criteria. Hence, the decision-maker can use the outcomes to reduce the risk of making a wrong decision and can compromise multi objectives in performance improvement.

Although the proposed framework used a public hospital in Thailand as the case study, it can be applied to other hospitals with similar characteristics for supporting decisions in resource management and customer satisfaction improvement. For further research, other optimization techniques can be applied to the model. Lexicographic optimizations use the order of setting objective functions according to their importance instead of giving weight and does not require normalized objective function [75]. Another method is goal programming which is more simplicity. The method requires the user to set the goal of each objective and optimizes the difference between goal and objective value [76]. The limitation of the study is the lack of timestamp data in the electronic system and the time that can collect the data is short.

## Figures and Tables

**Fig. 7:**
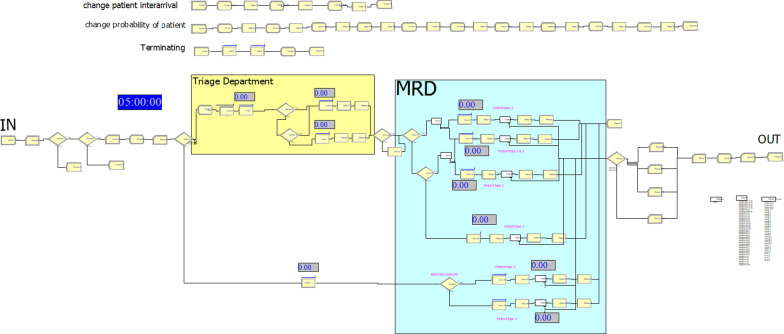
The simulation model in Arena simulation program

## Data Availability

The data that support the findings of this study are available from Thammasat University Hospital but restrictions apply to the availability of these data, which were used under license for the current study, and so are not publicly available. Data are however available from the authors upon reasonable request and with permission of Thammasat University Hospital.

## Appendix 1

The triangular distribution is used to fit the patient satisfaction data characteristics. The triangular distribution is a probability distribution with the lower limit (*a*), the upper limit (*b*), and the mode (*c*), presented as *Triangular(a,b,c)*. It has the mean ($\mu$) and SD ($\sigma$) equations presented in Eqs. 10 and 11, respectively [35].

10 $$\mu = \frac{a + b + c}{3}$$

11 $$\sigma = \frac{{\sqrt {\left( {a - b} \right)^{2} + \left( {a - c} \right)^{2} + \left( {b - c} \right)^{2} } }}{6}$$

## Appendix 2

The simulation model in Arena simulation program is shown in Fig. 7.

## Abbreviations

ANOVA: Analysis of variance
DES: Discrete event simulation
LOS: Length of stay
MR: Medical record
MRD: Medical record department
OPD: Outpatient department
TUH: Thammasat University Hospital

## References

[1] Gerfin M. Health Insurance and the Demand for Healthcare. In: Oxford Research Encyclopedia of Economics and Finance. 2019 Mar 26.

[2] Fadhil NF, Jusop M, Abdullah AA (2012). Hospital information system (his) implementation in a public hospital: a case study from malaysia. Far East J Psychol Bus.

[3] Ghazali RJ, Abd Manaf NH, Abdullah AH, Bakar AA, Salikin F, Umapathy M, Ali R, Bidin N, Ismail WI. Hospital waiting time: the forgotten premise of healthcare service delivery? Int J Health Care Quality Assur. 2011. 10.1108/09526861111160553 22204085

[4] Nottingham QJ, Johnson DM, Russell RS (2018). The effect of waiting time on patient perceptions of care quality. Qual Manag J.

[5] Tehrani AB, Feldman SR, Camacho FT, Balkrishnan R (2011). Patient satisfaction with outpatient medical care in the United States. Health Outcomes Res Med.

[6] Aburayya A, Alshurideh M, Albqaeen A, Alawadhi D, Ayadeh I (2020). An investigation of factors affecting patients waiting time in primary health care centers: an assessment study in Dubai. Manag Sci Lett.

[7] World Health Organization. Health workforce requirements for universal health coverage and the sustainable development goals (Human Resources for Health Observer, 17). World Health Organization. 2016. https://apps.who.int/iris/handle/10665/250330

[8] Obamiro JK (2013). Effects of waiting time on patient satisfaction: Nigerian hospitals experience. Int J Econ Behav.

[9] Dat LT. Associations between waiting time and patient satisfaction level at Tan Phu District Hospital in Ho Chi Mihn City, Vietham.

[10] Shaikh M, Miraldo M, Renner AT (2018). Waiting time at health facilities and social class: evidence from the Indian caste system. PLoS ONE.

[11] Lailomthong N, Prichanont S. Patient’s waiting time reduction in outpatient department. In: International conference on advances in engineering and technology ICAES; 2014 Mar 29, pp. 468–73.

[12] Greasley A, Owen C. Modelling people’s behaviour using discrete-event simulation: a review. Int J Oper Prod Manag. 2018.

[13] Freebairn L, Atkinson JA, Kelly PM, McDonnell G, Rychetnik L (2018). Decision makers’ experience of participatory dynamic simulation modelling: methods for public health policy. BMC Med Inform Decis Mak.

[14] Vieira B, Demirtas D, Van De Kamer JB, Hans EW, Van Harten W (2019). Improving workflow control in radiotherapy using discrete-event simulation. BMC Med Inform Decis Mak.

[15] Ortiz-Barrios M, Lopez-Meza P, McClean S, Polifroni-Avendaño G. Discrete-event simulation for performance evaluation and improvement of gynecology outpatient departments: a case study in the public sector. In: International conference on human-computer interaction; 2019, Springer, Cham, pp. 101–12.

[16] Saadouli H, Ltaif A (2021). Evaluating the impact of human resource management on the patient flow at an outpatient orthopedic clinic. Int J Healthc Manag.

[17] Silva SN, Hewapathirana RH, Jayatilleke WM (2017). Using a simulation modelling approach to manage outpatient department waiting time at the National Hospital of Sri Lanka. Stud Health Technol Inform..

[18] Eilers GM (2004). Improving patient satisfaction with waiting time. J Am Coll Health.

[19] Sun J, Lin Q, Zhao P, Zhang Q, Xu K, Chen H, Hu CJ, Stuntz M, Li H, Liu Y (2017). Reducing waiting time and raising outpatient satisfaction in a Chinese public tertiary general hospital-an interrupted time series study. BMC Public Health.

[20] Jenkinson C, Coulter A, Bruster S, Richards N, Chandola T (2002). Patients’ experiences and satisfaction with health care: results of a questionnaire study of specific aspects of care. Qual Saf Health Care.

[21] Ahmad I, Nawaz A, Khan S, Khan H, Rashid MA, Khan MH. Predictors of patient satisfaction. Gomal J Med Sci. 2011;9(2).

[22] Schoenfelder T, Klewer J, Kugler J (2011). Determinants of patient satisfaction: a study among 39 hospitals in an in-patient setting in Germany. Int J Qual Health Care.

[23] Aharony L, Strasser S (1993). Patient satisfaction: what we know about and what we still need to explore. Med Care Rev.

[24] Bjertnaes OA, Sjetne IS, Iversen HH (2012). Overall patient satisfaction with hospitals: effects of patient-reported experiences and fulfilment of expectations. BMJ Qual Saf.

[25] Simsekler MC, Alhashmi NH, Azar E, King N, Luqman RA, Al MA (2021). Exploring drivers of patient satisfaction using a random forest algorithm. BMC Med Inform Decis Mak.

[26] Almeida RS, Bourliataux-Lajoinie S, Martins M (2015). Satisfaction measurement instruments for healthcare service users: a systematic review. Cad Saude Publica.

[27] Al-Abri R, Al-Balushi A (2014). Patient satisfaction survey as a tool towards quality improvement. Oman Med J.

[28] O’Leary KJ, Killarney A, Hansen LO, Jones S, Malladi M, Marks K, Shah HM (2016). Effect of patient-centred bedside rounds on hospitalised patients’ decision control, activation and satisfaction with care. BMJ Qual Saf.

[29] Hof M, Tepper G, Semo B, Arnhart C, Watzek G, Pommer B (2014). Patients' perspectives on dental implant and bone graft surgery: questionnaire-based interview survey. Clin Oral Implant Res.

[30] Boudreaux ED, O'Hea EL (2004). Patient satisfaction in the emergency department: a review of the literature and implications for practice. J Emerg Med.

[31] Larsen DL, Attkisson CC, Hargreaves WA, Nguyen TD (1979). Assessment of client/patient satisfaction: development of a general scale. Eval Program Plann.

[32] Shirley ED, Sanders JO (2013). Patient satisfaction: implications and predictors of success. JBJS.

[33] Thompson DA, Yarnold PR, Williams DR, Adams SL (1996). Effects of actual waiting time, perceived waiting time, information delivery, and expressive quality on patient satisfaction in the emergency department. Ann Emerg Med.

[34] Xie Z, Or C (2017). Associations between waiting times, service times, and patient satisfaction in an endocrinology outpatient department: a time study and questionnaire survey. INQUIRY J Health Care Organ Provis Financ..

[35] Anderson RT, Camacho FT, Balkrishnan R (2007). Willing to wait? The influence of patient wait time on satisfaction with primary care. BMC Health Serv Res.

[36] Fan X, Tang J, Yan C, Guo H, Cao Z (2019). Outpatient appointment scheduling problem considering patient selection behavior: data modeling and simulation optimization. J Comb Optim.

[37] Yin C, McKay A. Introduction to modeling and simulation techniques. In: Proceedings of ISCIIA 2018 and ITCA 2018; 2018, Leeds.

[38] van Lent WA, VanBerkel P, van Harten WH (2012). A review on the relation between simulation and improvement in hospitals. BMC Med Inform Decis Mak.

[39] Leemis LM, Park SK (2006). Discrete-event simulation: a first course.

[40] Bhattacharjee P, Ray PK (2014). Patient flow modelling and performance analysis of healthcare delivery processes in hospitals: a review and reflections. Comput Ind Eng.

[41] Kovalchuk SV, Funkner AA, Metsker OG, Yakovlev AN (2018). Simulation of patient flow in multiple healthcare units using process and data mining techniques for model identification. J Biomed Inform.

[42] Devapriya P, Strömblad CT, Bailey MD, Frazier S, Bulger J, Kemberling ST, Wood KE (2015). StratBAM: a discrete-event simulation model to support strategic hospital bed capacity decisions. J Med Syst.

[43] Luo L, Liu H, Liao H, Tang S, Shi Y, Guo H (2016). Discrete event simulation models for CT examination queuing in West China Hospital. Comput Math Methods Med.

[44] Bartos BJ, Mioduszewski M, Renner M, McCleary R. An application of discrete event simulation for planning and resource allocation in a state hospital system servicing both criminal and civil commitments. In: 2017 Winter simulation conference (WSC); 2017, IEEE, pp. 4509–511.

[45] Kritchanchai D, Hoeur S (2018). Simulation modeling for facility allocation of outpatient department. Int J Healthc Manag.

[46] Kalwar MA, Mari SI, Memon MS, Tanwari A, Siddiqui AA (2020). Simulation based approach for improving outpatient clinic operations. Mehran Univ Res J Eng Technol.

[47] Kittipittayakorn C, Ying KC (2016). Using the integration of discrete event and agent-based simulation to enhance outpatient service quality in an orthopedic department. J Healthc Eng..

[48] Baril C, Gascon V, Miller J, Côté N (2016). Use of a discrete-event simulation in a Kaizen event: a case study in healthcare. Eur J Oper Res.

[49] Gul M, Guneri AF (2015). A comprehensive review of emergency department simulation applications for normal and disaster conditions. Comput Ind Eng.

[50] Lin WD, Chia L. Combined forecasting of patient arrivals and doctor rostering simulation modelling for hospital emergency department. In: 2017 IEEE International conference on industrial engineering and engineering management (IEEM); 2017, IEEE, pp. 2391–395.

[51] Schmidt R, Geisler S, Spreckelsen C (2013). Decision support for hospital bed management using adaptable individual length of stay estimations and shared resources. BMC Med Inform Decis Mak.

[52] Neumann J, Angrick C, Höhn C, Zajonz D, Ghanem M, Roth A, Neumuth T (2020). Surgical workflow simulation for the design and assessment of operating room setups in orthopedic surgery. BMC Med Inform Decis Mak.

[53] Wang Y, Lee LH, Chew EP, Lam SS, Low SK, Ong ME, Li H. Multi-objective optimization for a hospital inpatient flow process via discrete event simulation. In: 2015 Winter simulation conference (WSC); 2015, IEEE, pp. 3622–631.

[54] Chen PS, Yang KH, Robielos RA, Cancino RA, Dizon LA (2016). Patient referral mechanisms by using simulation optimization. Simul Model Pract Theory.

[55] Chen PS, Lin MH (2017). Development of simulation optimization methods for solving patient referral problems in the hospital-collaboration environment. J Biomed Inform.

[56] Ibrahim IM, Liong CY, Bakar SA, Ahmad N, Najmuddin AF. Minimizing patient waiting time in emergency department of public hospital using simulation optimization approach. In: AIP conference proceedings, vol. 1830, no. 1; 2017 Apr 27, AIP Publishing LLC, p. 060005.

[57] Chen TL, Wang CC (2016). Multi-objective simulation optimization for medical capacity allocation in emergency department. J Simul.

[58] Keshtkar L, Salimifard K, Faghih N (2015). A simulation optimization approach for resource allocation in an emergency department. QScience Connect.

[59] Bouajaja S, Dridi N (2017). A survey on human resource allocation problem and its applications. Oper Res Int J.

[60] Petering ME, Aydas OT, Kuzu K, Ross A (2015). Simulation analysis of hospital intensive care unit reimbursement policies from the triple bottom line perspective. J Simul.

[61] Aliyu AI, Sulaiman TA, Yusuf A (2015). Modeling and simulation analysis of health care appointment system using ARENA. Int J.

[62] Chang J, Zhang L (2019). Case Mix Index weighted multi-objective optimization of inpatient bed allocation in general hospital. J Comb Optim.

[63] Software V, Software V. Vose Software [Internet]. Vosesoftware.com. 2021 [cited 29 November 2021]. Available from: https://www.vosesoftware.com/riskwiki/distributionsusedinmodelingexpertopinion.php.

[64] Banks J. Discrete event system simulation. Pearson Education India; 2005.

[65] Naylor TH, Finger JM (1967). Verification of computer simulation models. Manag Sci.

[66] Marler RT, Arora JS (2004). Survey of multi-objective optimization methods for engineering. Struct Multidiscip Optim.

[67] Coello CA, Christiansen AD (1995). An approach to multiobjective optimization using genetic algorithms. Intell Eng Syst Through Artif Neural Netw.

[68] Zimmermann HJ (1978). Fuzzy programming and linear programming with several objective functions. Fuzzy Sets Syst.

[69] Lin CC (2004). A weighted max–min model for fuzzy goal programming. Fuzzy Sets Syst.

[70] Amid A, Ghodsypour SH, O’Brien C (2011). A weighted max–min model for fuzzy multi-objective supplier selection in a supply chain. Int J Prod Econ.

[71] Montgomery DC, Runger GC, Hubele NF (2009). Engineering statistics.

[72] Tukey JW. Exploratory data analysis. 1977.

[73] Kelton WD, Sadowski RP, Sturrock DT. Simulation with Arena with CDROM.

[74] Warm Up Time [Internet]. Simul8.com. 2021 [cited 30 November 2021]. Available from: https://www.simul8.com/support/help/doku.php?id=gettingstarted:techguide:warmup.

[75] Arora JS. Multiobjective optimum design concepts and methods. In: Introduction to optimum design. 2012, pp. 657–79.

[76] Tamiz M, Jones DF, El-Darzi E (1995). A review of goal programming and its applications. Ann Oper Res.

